# Posterior Spinal Cord Infarction Complicating a Bronchial Arterial Embolization

**DOI:** 10.7759/cureus.73594

**Published:** 2024-11-13

**Authors:** Daniela Barbosa Mateus, Antony Dionisio, Tânia F Mendes, Ana Margarida Araújo, João Gonçalves Pereira

**Affiliations:** 1 Department of Internal Medicine, Hospital Vila Franca de Xira, Vila Franca de Xira, PRT; 2 Department of Internal Medicine, Hospital São Francisco Xavier, Lisbon, PRT; 3 Intensive Care Unit, Hospital Vila Franca de Xira, Vila Franca de Xira, PRT

**Keywords:** bronchial arterial embolization, hemoptysis, posterior spinal cord infarction, pulmonary hypertension, pulmonary veno-occlusive disease

## Abstract

Massive hemoptysis is a life-threatening condition. Bronchial artery embolization (BAE) is an effective technique for controlling bleeding in cases of severe hemoptysis, with infrequent complications. While rare, spinal cord infarction is a serious potential complication of BAE.

Here, we present a case involving a 28-year-old man with idiopathic pulmonary hypertension who underwent BAE after recurrent severe hemoptysis. Following the procedure, he developed urinary retention and progressive sensory deficits, culminating in significant motor impairment. Magnetic resonance imaging (MRI) revealed ischemic lesions in the posterior spinal cord, resulting in a diagnosis of iatrogenic spinal cord ischemia.

While BAE is an effective therapeutic option for severe hemoptysis, it carries the risk of serious complications, including spinal cord ischemia. This case underscores the potential for iatrogenic spinal cord injury following BAE and highlights the need for increased awareness in high-risk patients.

## Introduction

Massive hemoptysis is a critical medical emergency often leading to severe morbidity and mortality if not promptly managed. Previous studies report in-hospital mortality after hemoptysis of 8-10% [1]. Bronchial artery embolization (BAE) effectively stops bleeding after severe hemoptysis, with rare complications. BAE has emerged as a first-line intervention for controlling severe hemoptysis, demonstrating effectiveness in halting bleeding while maintaining a long-term survival rate of approximately 85% [2-4]. Although rare, dramatic consequences of this procedure can occur, such as spinal cord infarction, resulting from the embolization of a spinal artery. Spinal cord ischemia, following BAE, is thought to arise from inadvertent embolization of radiculomedullary arteries, which supply critical spinal cord regions. The prevalence of such complications, although low, underscores the necessity for vigilance in the management of patients undergoing BAE, particularly those with underlying vascular anomalies or other complicating factors [3].

We report a case of a young patient with idiopathic pulmonary hypertension who was admitted for recurrent hemoptysis. As part of the treatment, he underwent BAE, which subsequently resulted in posterior spinal cord infarction as a complication.

## Case presentation

A 28-year-old man presented to the emergency room with recurrent severe episodes of hemoptysis. His clinical history was remarkable for pulmonary hypertension, secondary to veno-occlusive disease diagnosed one year early. He was treated with ambrisentan (10mg daily) and tadalafil (20mg daily). Rivaroxaban (for a possible past pulmonary embolism) had recently been discontinued due to recurrent hemoptysis.

On admission, the patient was alert, cooperative, and eupneic. His blood pressure was normal. A laboratory test excluded a drop in hemoglobin concentration or coagulopathy. Flexible bronchoscopy revealed blood in both main bronchi, mainly in the left lower lobe bronchus and right upper lobe bronchus, but no active bleeding was noted. Despite treatment with intravenous and inhaled tranexamic acid, as well as intravenous aminocaproic acid, the hemoptysis persisted.

The patient was transferred to another hospital to undergo bronchial arterial embolization, under general anesthesia. Angiography, by right femoral artery access, revealed bilateral diffuse arterial hypertrophy in the lungs. Superselective catheterization and embolization with arterial exclusion were performed, with adequate vascular results.

On the next day, the patient noted urinary retention and dysesthesia in the lower limbs. His condition rapidly progressed to include fecal incontinence and decreased sensitivity, with a D5 sensitive level. Additional neurological examination revealed no deficits in cranial nerve functions, a negative pronator drift test, and a positive Mingazzini test, with gradual right-sided limb descent, eventually hitting the bed. There was asymmetrical paresis, with muscle strength graded 4/5 in the right lower limb and 4+/5 in the left lower limb. Significant spasticity was noted, particularly in the right lower limb. Sensory examination showed hypoesthesia, with loss of pain sensation at the right D4 and the left D8-9 dermatomes. Dysesthesia was present to light touch, primarily affecting the distal lower limbs. The Lhermitte sign was negative, and the Babinski sign was present bilaterally.

**Figure 1:**
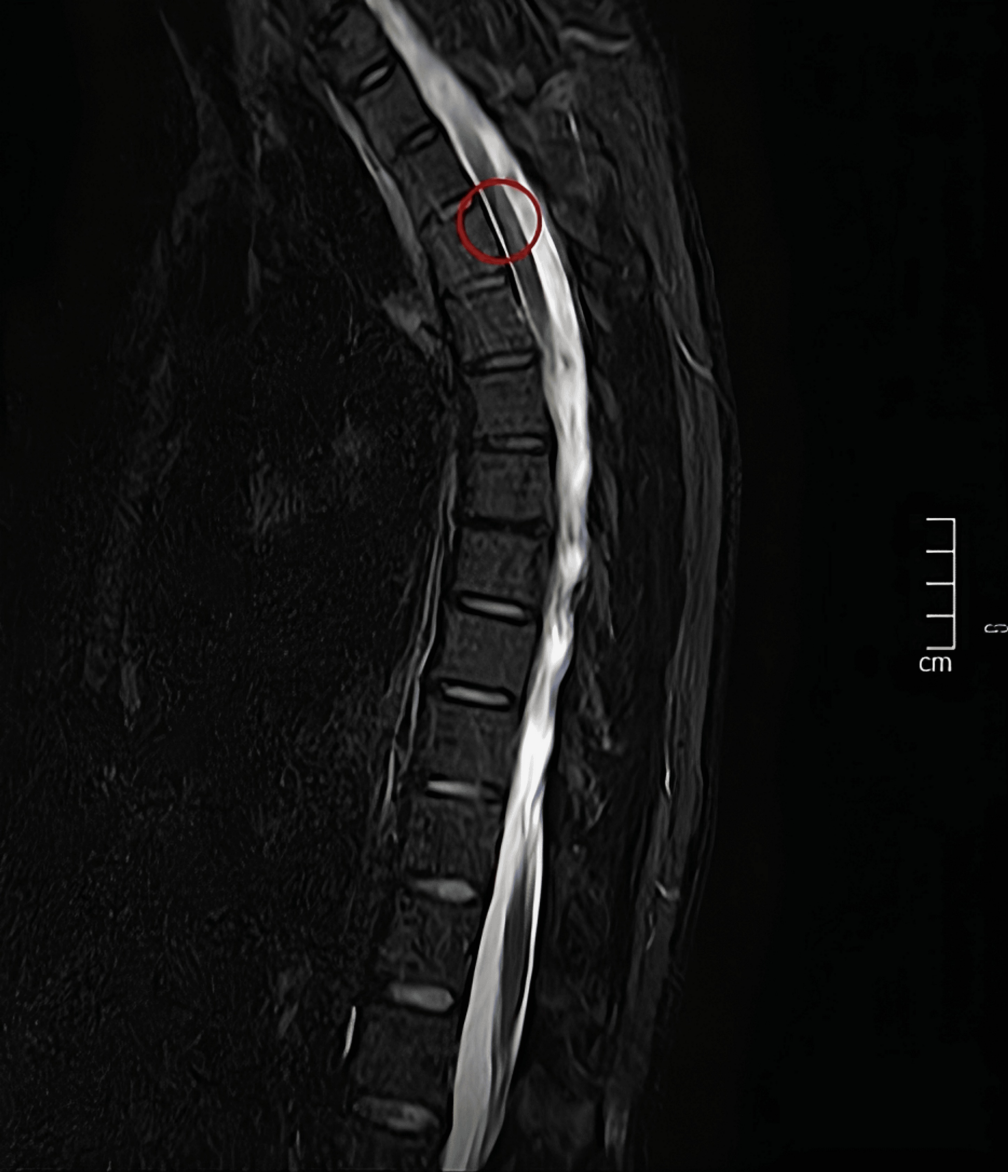
STIR sagittal plane of the dorsal spin. Several focal lesions are observed in the dorsal cord, longitudinally short, without significant cord swelling, and no evidence of bone infarcts in the vertebrae of this segment, likely to correspond to spinal cord infarcts in the posterior spinal artery system STIR: Short tau inversion recovery

**Figure 2:**
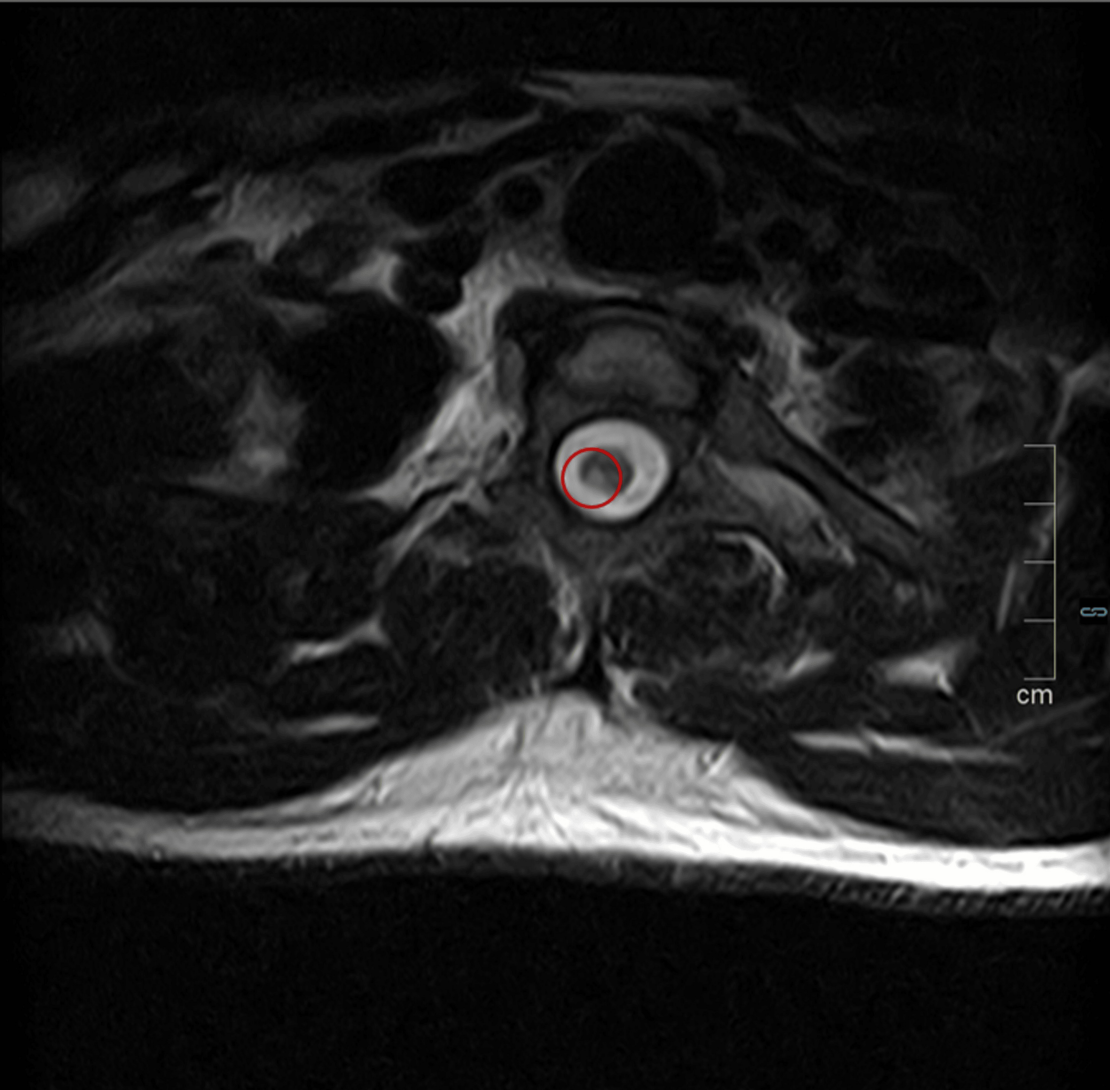
Axial T2 plane of the dorsal spine. Lesions are focused on the posterior columns, with right-sided laterality (compatible with the territory of the right posterior spinal artery)

Magnetic resonance imaging (MRI) of the brain and spine demonstrated ischemic lesions, predominantly in the posterior and lateral segments of the spinal cord, between D2 and D5. No gadolinium enhancement was noted. These aspects were deemed suggestive of an ischemic or demyelinating pathology (Figures 1, 2). Given the temporal relationship to the embolization procedure, iatrogenic spinal cord ischemia was diagnosed.

Evaluation of a cerebrospinal fluid (CSF) specimen excluded pathological findings (Table 1). Due to the bleeding risk, antithrombotic therapy was withheld.

**Table 1:** Laboratory evaluation of a CSF sample. CSF: Cerebrospinal fluid, ANA: Antinuclear antibodies; anti.dsDNA: Antibodies to double-stranded, ANCAs: Antineutrophil cytoplasmic antibodies; c-ANCA: Cytoplasmic antineutrophil cytoplasmic antibodies; Anti-MOG: Antibodies to myelin oligodendrocyte glycoprotein

CSF Results	Value	Reference Range
Oligoclonal bands	Absent	-
Virus panel (herpes, CMV, EBV, VZV, enterovirus)	Negative	-
VZV Ag IgG	Negative	-
VDRL	Negative	-
Glucose	59 mg/dL	>40 mg/dL
Proteins	48 mg/dL	15-45 mg/dL
Chlorides	122 mEq/L	116-127 mEq/L
White cells	1 cel/uL -mononuclear	<5
Bacteriological CSF	Negative	-
Autoimmunity (ANA, anti-dsDNA, ANCAs, c-ANCA, anti-MOG)	Negative	-

Throughout hospitalization, the patient’s neurological status improved gradually. At the time of discharge, 24 days after admission, he exhibited residual spasticity in the lower limbs (grade 3) and sensory deficits, with hypoesthesia to tactile and pain stimuli at the D4 dermatome level on the right, and L4 dermatome level on the left. Motor function in the upper limbs remained intact.

## Discussion

We report a case of a spinal cord infarct, as a complication of BAE performed to control recurrent hemoptysis. BAE is a successful and usually safe procedure. Allowing the exclusion of a bleeding artery in the tracheal and bronchial tree and the resolution of recurrent or even massive hemoptysis. Complications of BAE are rare but often severe: aortic dissection, mediastinal hematoma, esophageal necrosis, systemic infarcts, and cerebral or spinal cord infarctions [2,4]. The spinal cord is supplied mainly by three arterial branches: the anterior spinal artery, responsible for the anterior two-thirds of the cord, and two posterior spinal arteries. At the cervical level, the radiculomedullary arteries originate from the vertebral arteries and supply the top part of the spinal cord [5]. In our patient, the extensive arterial hypertrophy likely contributed to a higher risk of radiculomedullary artery involvement during embolization, leading to ischemic injury to the spinal cord.

**Figure 3:**
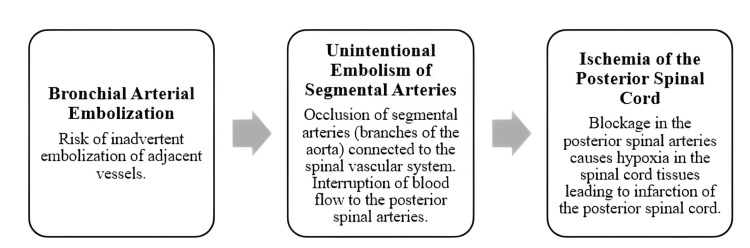
Diagram of the spinal cord injury mechanism Image Credits: Daniela Barbosa Mateus

Neurological complications may occur as a complication of BAE, mainly due to inadvertent embolization of foreign material [2,5]. Anterior spinal cord infarction is the most common. Posterior infarction is rare because of the anastomotic network of direct penetrating vessels and a plexus of pial vessels fed by the paired posterior spinal artery (Figure 3).

The differential diagnosis of spinal cord ischemia includes transverse myelitis and demyelinating diseases. In our patient, the MRI findings, the absence of pathological changes in the CSF, and the temporal relationship supported the diagnosis of an iatrogenic ischemia.

This case underscores the challenges of treating patients with rare and complex diseases. Pulmonary veno-occlusive disease often requires a delicate balance between managing anticoagulation for thromboembolic risk and controlling bleeding complications, like hemoptysis [6]. Although BAE is effective for controlling bleeding, it is not without risks, particularly in patients with complex vascular anatomies.

## Conclusions

BAE is an effective therapeutic option for severe hemoptysis. While BAE has demonstrated efficacy in controlling life-threatening bleeding, this case underscores the potential for serious complications, such as spinal cord ischemia. This complication may arise from technical factors, inadvertent embolization into spinal arteries, or unclear anastomotic connections between the bronchial and spinal circulations. Therefore, continuous monitoring and thorough post-procedural assessments are critical for the prompt identification and management of any emerging complications.
